# Measurement of sialic acid content on recombinant membrane proteins

**DOI:** 10.1186/1753-6561-5-S8-P59

**Published:** 2011-11-22

**Authors:** Deniz Baycin-Hizal, Sunny Mai, Daniel Wolozny, Ilhan Akan, Noboru Tomiya, Karen Palter, Michael Betenbaugh

**Affiliations:** 1 Department of Chemical and Biomolecular Engineering, Johns Hopkins University, Baltimore, MD, 21218, USA; 2 Department of Biology, Temple University, Philadelphia, PA, 19122, USA; 3 Department of Biology, Johns Hopkins University, Baltimore, MD, 21218, USA

## Background

Membrane proteins such as cell adhesion molecules, receptors, transporters and ion channel proteins all have essential roles in cell-growth, migration, and flow of information, cell-cell and cell-protein communication. Membrane proteins are targets of biopharmaceutical companies because they have diverse effects on the progression of many diseases [1]. Ion channels are membrane proteins that play critical roles in a number of cell functions including communication and neuromuscular activity. Treatment of channelopathy diseases such as cancer, cardiac arrhythmia, ataxia, paralysis, epilepsy, memory and learning loss, requires a broad understanding of ion channel function. Sialic acid is a critical charged glycan that affects the action potential of potassium channels which leads to changes in the neuronal system of organisms. In order to understand the effect of the sialylation on channel function, the presence of the sialic acid on the protein of interest should be studied. In this study, a novel method for the quantification of sialic acid is described for a potassium channel membrane protein.

## Materials and methods

### Cell lines

HEK293 cells were grown in DMEM media (Invitrogen, Carlsbad, CA) supplemented with 10% Fetal Bovine Serum (Invitrogen), 1% nonessential amino acids (Invitrogen) and 1% L-Glutamine (Invitrogen).

### Transfection

Lipofectamine^TM^ 2000 Reagent (Invitrogen) was used to transfect potassium channel into HEK293 cells. Stable pools were formed after 15 days of antibiotic selection and 8 different clones were selected from the pools.

### Western blot analysis

Cells were lysed in RIPA buffer containing complete-mini EDTA free protease inhibitor cocktail (Roche Diagnostics, Mannheim, Germany). The protein concentrations of each sample were determined by using a BCA™ protein assay kit (Pierce, Rockford, IL). Equal total protein amounts were loaded onto 8% gels and separated by electrophoresis. The separated proteins were transferred from the gel to membrane. For detection of the channel protein, membrane was incubated in 1% milk in PBST solution containing primary antibody with a dilution of 1:1000 overnight at 4°C. A secondary anti-rabbit IgG HRP conjugate antibody (Amersham, Louisville, CO) was used at a dilution of 1:5000 in 1% milk/PBST overnight at 4°C.

### Immunoprecipitation

The potassium channel protein was purified by using G-beads coupled antibody.

### HPLC analysis

The purified protein was run on the gel and protein band was cut from the gel. The cut band was acid hydrolyzed to release the sialic acid. The released sialic acid was derivatized with fluorescent 4,5-Methylenedioxy-1,2-phenylenediamine dihydrochloride (DMB) reagent and injected to HPLC [2].

## Results

**Figure 1:**
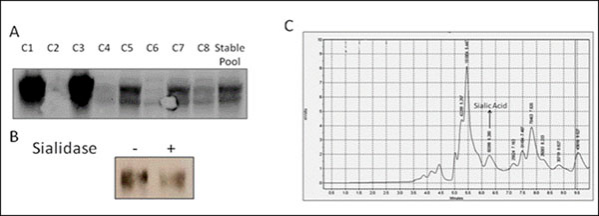
Potassium channel sialylation. A) Western blot analysis of channel proteins B) Western blot of the channel protein before and after sialidase treatment C) Sialic acid presence in HPLC.

The transient expression of potassium channel was evaluated by western blot analysis. After 15 days of antibiotic selection, an expression level of the gene of interest was evaluated by Western blot. The stable pool expression is an average of the varied expression levels of the protein of interest in cells. In order to decrease the expression heterogeneity and ensure that all the cells contain the same genetic content, eight different single clonal isolates were selected. The quality and quantity of the protein expression in these clonal isolates was compared with the protein expression of stable pool. Figure 1.A shows the western blot of 8 cell clones and stable pool of potassium channel. Both glycosylated and non-glycosylated bands of the specific protein can be distinguished in the Western blot. Although some of the clones and stable pool results showed non-glycosylated bands and poor expression, clone 1 and 3 showed high glycosylation quality and quantity. For this reason, clone 3 was scaled up and purified using immunoprecipitation. The purified protein was treated with sialidase and the mobility shift of the protein before sialidase and after sialidase treatment was compared by running Western blot as shown in Figure 1.B. Due to the low molecular weight of sialic acid, the mobility shift detected by Western blot was minimal. In order to quantify the amount of sialic acid found on the protein, about 6 pmol of purified protein was run on the SDS-PAGE gel and was cut from the gel. The cut band was acid hydrolyzed to release the sialic acid and the released sialic acid was derivatized with DMB reagent and injected into HPLC. The sialic acid amount that is released from the gel was calculated by using a sialic acid calibration curve (data not shown). The amount of sialic acid was calculated to be 19 pmol depending on HPLC and calibration curve results. Since the potassium channel of interest may have 4 possible sites for the sialylation, and therefore 24 pmols sialic acid. For this reason, the estimated sialic acid occupancy efficiency of the channel was found as 79%.

## Conclusion

Membrane proteins are to be very difficult to produce and purify for structural or glycan analysis because of their low expression levels. In this study, HEK 293 cells were used to express a potassium channel protein and clonal isolates were picked from stable pools to evaluate the quality and quantity of the glycosylated protein. A highly expressing clone with glycosylation was then selected for the purification and sialic acid analysis. The results showed that the sialic acid occupancy efficiency of the channel of interest was around 80% when expressed in HEK293 cells.
